# Surface Markers and Chemokines/Cytokines of Tumor-Associated Macrophages in Osteosarcoma and Other Carcinoma Microenviornments—Contradictions and Comparisons

**DOI:** 10.3390/cancers16162801

**Published:** 2024-08-08

**Authors:** Rikito Tatsuno, Yoshihiro Komohara, Cheng Pan, Tomonori Kawasaki, Atsushi Enomoto, Takahiro Jubashi, Hiroyuki Kono, Masanori Wako, Tomoyuki Ashizawa, Hirotaka Haro, Jiro Ichikawa

**Affiliations:** 1 Department of Orthopaedic Surgery, University of Yamanashi, Yamanashi 400-0016, Japan; rtatsuno@yamanashi.ac.jp (R.T.); tjubashi@yamanashi.ac.jp (T.J.); hkono@yamanashi.ac.jp (H.K.); wako@yamanashi.ac.jp (M.W.); tashizawa@yamanashi.ac.jp (T.A.); haro@yamanashi.ac.jp (H.H.); 2 Department of Cell Pathology, Graduate School of Medical Sciences, Kumamoto University, Kumamoto 860-8555, Japan; ycomo@kumamoto-u.ac.jp (Y.K.); tschpuhn@kumamoto-u.ac.jp (C.P.); 3 Department of Pathology, Saitama Medical University International Medical Center, Saitama 350-1298, Japan; tomo.kawasaki.14@gmail.com; 4 Department of Pathology, Graduate School of Medicine, Nagoya University, Nagoya 464-8601, Japan; enomoto@iar.nagoya-u.ac.jp

**Keywords:** osteosarcoma, tumor microenvironment, tumor-associated macrophages, surface marker, chemokine, cytokine

## Abstract

**Simple Summary:**

Osteosarcoma (OS) is the most frequently occurring malignant bone tumor in children. Although advances in chemotherapy and surgery have gradually improved OS prognosis, no improvement has been reported over the past two decades. Recently, tumor microenvironment (TME) has attracted attention as a novel therapeutic target. The TME includes peritumoral immune cells, blood vessels, extracellular matrix, fibroblasts, lymphocytes, bone marrow-derived inflammatory cells, platelets, and signaling molecules, which create an environment that promotes tumor growth, metastasis, and anticancer drug resistance. Research on the TME is particularly important because tumor-associated macrophages (TAMs) are a major component of this microenvironment, and the interaction between tumors and TAMs contributes towards tumor aggressiveness. However, our knowledge of the interaction between OS and TAMs is limited. In this review, we aim to describe the characteristics of TAMs in the OS TME.

**Abstract:**

Osteosarcoma (OS) is the most common primary bone tumor in children and adolescents. Prognosis is improving with advances in multidisciplinary treatment strategies, but the development of new anticancer agents has not, and improvement in prognosis for patients with pulmonary metastases has stalled. In recent years, the tumor microenvironment (TME) has gained attention as a therapeutic target for cancer. The immune component of OS TME consists mainly of tumor-associated macrophages (TAMs). They exhibit remarkable plasticity, and their phenotype is influenced by the TME. In general, surface markers such as CD68 and CD80 show anti-tumor effects, while CD163 and CD204 show tumor-promoting effects. Surface markers have potential value as diagnostic and prognostic biomarkers. The cytokines and chemokines produced by TAMs promote tumor growth and metastasis. However, the role of TAMs in OS remains unclear to date. In this review, we describe the role of TAMs in OS by focusing on TAM surface markers and the TAM-produced cytokines and chemokines in the TME, and by comparing their behaviors in other carcinomas. We found contrary results from different studies. These findings highlight the urgency for further research in this field to improve the stalled OS prognosis percentages.

## 1. Introduction

Osteosarcoma (OS) is the most common primary bone tumor in children and adolescents. Prior to the 1970s, OS treatment primarily consisted of limb-sparing surgery or amputation, with a 5-year survival rate of 20% [1]. Subsequently, with the introduction of chemotherapy, the survival rates for patients with localized OS increased to approximately 70% [2]. However, the prognosis for patients with metastases remains poor at 20% to 30% [3]. Thus, there is a need to elucidate the mechanisms of pulmonary metastasis and to develop novel therapeutic agents.

Current OS treatment consists of preoperative chemotherapy, surgery, and postoperative chemotherapy using high-dose methotrexate, doxorubicin, cisplatin (MAP), ifosfamide, etoposide, cyclophosphamide, and carboplatin. Although the combination of drugs with different mechanisms of action increases the overall therapeutic efficacy, it causes a high rate of complications such as renal and liver damage and bone marrow suppression [4]. It is believed that using a combination of novel anticancer agents with current therapies would result in (i) additive synergistic effects, (ii) reduced toxicity due to dose reduction, and (iii) application to metastatic cases with particularly low response rates. However, as observed in recent years in carcinoma research, no new anticancer agents are being developed presently.

In recent years, the tumor microenvironment (TME) has gained attention as a therapeutic target for cancer; the TME includes innate and adaptive immune cells, stromal and endothelial cells, cancer-associated fibroblasts, and the extracellular matrix. Together, these components form a niche in which tumor cells can grow and seed. The immune component of the OS microenvironment consists primarily of tumor-associated macrophages (TAMs) [5]. The cytokines and chemokines produced by TAMs promote tumor growth, metastasis promotion, and angiogenesis. TAMs express surface markers such as CD163 and CD204, which have potential value as diagnostic and prognostic biomarkers [6,7,8,9].

The role of TAMs in OS remains poorly understood. The purpose of this review was to provide a comprehensive description of TAMs in OS. We have focused on TAM surface markers and the TAM-associated cytokines/chemokines in OS and compared their similarities and differences with other carcinomas.

## 2. Tumor Microenvironment (TME) of OS

In addition to OS cells, the TME of OS includes osteoclasts, tumor-infiltrating lymphocytes including T and NK cells as immune cells, myeloid cells such as dendritic cells, and macrophages, fibroblasts, pericytes, mesenchymal stem cells, myoblasts, endothelial cells, and others, thereby forming a complex network [10]. In the OS TME, platelets [11,12], neutrophils [13], and mesenchymal stem cells [14,15] play an important role in tumor progression.

A large percentage of the immune cells in the TME are TAMs, which produce a variety of humoral factors and promote tumor-cell proliferation, metastasis, invasion, angiogenesis, immunosuppressive effects, and immune escape [16,17].

### M1/M2 Macrophages

In the 1990s, Stein et al. reported that macrophages activated by interleukin (IL)-4 through its unique pathway show a different activation phenotype compared to that observed in macrophages activated by other stimulants [18]. Later, Mills et al. proposed the concept of M1 and M2 macrophages [19]. M1 macrophages are stimulated by IFN-γ and bacterial components such as lipopolysaccharide (LPS), Th1 response-related interferons (IFN-α/β), IFN-γ, and Toll-like receptor ligands, which are responsible for killing bacteria [20]. In contrast, M2 macrophages are induced by anti-inflammatory factors such as transforming growth factor (TGF)-β and glucocorticoids, IL-4, IL-10, and IL-13, which have anti-inflammatory effects and promote tissue repair [21,22]. Thus, the concept of a single type of macrophage oscillating between two states (polarization) was proposed. [23].

## 3. Tumor-Associated Macrophages (TAMs)

TAMs are another one of the diverse macrophage subtypes. They exhibit remarkable plasticity, and their phenotype is influenced by the TME. In several carcinomas, M1 TAMs have anti-tumor effects, whereas M2 TAMs have tumor-promoting effects [24]. Representative pan TAM markers include CD11b [25], F4/80 [26] and Iba-1 [27]; M1-TAM markers include CD68 [28], CD80 [29], CD86 [30], and inducible nitric oxide synthase (iNOS) [31]; and M2-TAM markers include CD163 [32], CD204 [9], CD206 [30], CD209 [33], and arginase-1 [32]. Here, we discuss the TAM surface markers that have been identified in OS (Table 1).

### 3.1. M1/M2-Related TAM Markers

#### 3.1.1. Iba-1

Iba1 (Ionized calcium-binding adapter molecule 1) is expressed specifically in microglia in the central nervous system and is used as a microglial marker. In peripheral tissues, it is expressed on macrophages and is also known as AIF-1 (Allograft inflammatory factor-1).

**Figure 1:**
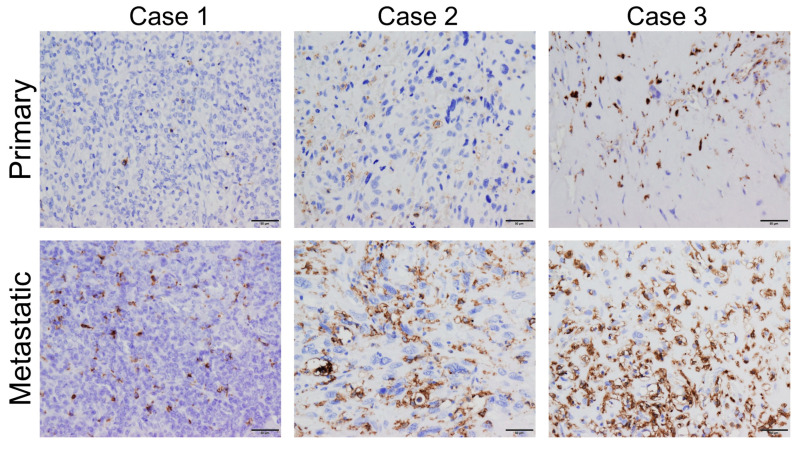
Cell-cell interaction between TAMs and OS cells. IHC analysis indicated that TAMs are predominant stromal cells in OS tissues, especially in metastatic lesions. Picture figures were IHC of Iba-1 (a pan-macrophage marker) from paired primary lesion and lung metastatic lesion of three cases. Scale bar: 50 mm. Picture figures of Iba1 IHC of three cases shown in our previous study were presented [34]. IHC was performed in newly prepared sections as described in a previous study [52]. The study was approved by the ethics committees of each institution (No. 2456).

Iba-1 was reported as a pan-TAM marker in carcinomas and undifferentiated pleomorphic sarcoma [48,49,50,51]. We previously compared TAM quantities in primary and lung metastatic OS sites in patients with lung metastases using immunohistochemistry and found a predominance of Iba-1 positive TAMs in lung metastases [34] (Figure 1). 

#### 3.1.2. iNOS

Nitric oxide (NO) produced by iNOS in M1 macrophages promotes tumor cell apoptosis. The iNOS gene expression is regulated via MAPK and NF-κB signaling pathways [53,54,55].

Low expression of iNOS in the intratumor region compared with that in the invasive front is associated with poor prognosis in colorectal cancer (CRC). Using the iNOS expression in combination with other markers (CXCL10 and CD11c) can help assess the prognosis of patients with CRC more accuracy [56].

In OS, the levels of iNOS were higher in patient samples without OS metastasis than in those with OS metastasis. Multivariate analysis shows that iNOS is a predictor of OS metastasis [35].

#### 3.1.3. CD80/CD86

CD80 and CD86 are expressed on antigen-presenting cells and bind to the homologous T-cell receptors CD28 and CTLA-4 to release co-stimulatory signals that are required for optimal T-cell activation [57].

TAMs obtained from patients with gastric cancer with peritoneal dissemination exhibit lower CD80 and CD86 expression compared with that in M1 macrophages induced by LPS and IFN-γ [58]. IL-6 secreted by CD80^+^ and CD86^+^ M1-like TAMs is reported to promote the progression of oral squamous cell carcinoma (OSCC) [59].

In OS, CD80^+^ TAM levels are significantly decreased in metastases compared to those in primary tumors, and decreased M1 TAMs may be associated with OS metastasis [36]. Anti-PD-1 therapy shifts CD163^+^ M2 TAMs to CD86^+^ M1 TAMs and inhibits lung metastases in OS [37]. CD86 expression is upregulated in OS and is associated with a favorable prognosis. It is also significantly correlated with naive B cells and M2 macrophages. Some reports suggest that CD86 is predominantly expressed on M2 macrophages because M0 and M2 macrophages are the major components of tumor-infiltrating immune cells in OS tissue [38].

#### 3.1.4. CD68

CD68, first identified as a KP1 monoclonal antibody, is a highly glycosylated glycoprotein, also known as GP110, LAMP4, or SCARD1, a 110 kDa transmembrane glycoprotein widely expressed on monocytic cell types including macrophages, microglia, and osteoclasts [60,61,62].

Regarding the expression levels of CD68 as a M1-TAM marker in carcinomas and prognosis, it has been reported that the low expression of CD68 and CD163 in classical Hodgkin lymphoma indicates a better prognosis [63]. In metastases of the lung and pancreas in OSCC, CD68^+^ TAMs are the main source of lipid droplet-associated PLIN2; high levels of PLIN2 may exacerbate the TNM stage and promote the malignant phenotype of OSCC [64]. In contrast, there are reports that CD68 expression levels in classical Hodgkin lymphoma do not influence the achievement of complete remission, progression-free survival, or disease-specific survival [65].

In OS, infiltration of CD68^+^ macrophages is associated with a better prognosis in patients with OS [39]; the total number of CD68 macrophages in patient samples with and without metastases was reported to be the same [35].

However, CD68 cross-reacts with mesenchymal cells [66] and is also considered a marker for osteoclasts [67,68]. Therefore, there is an opinion that CD68 is not suitable as an M1-TAM marker in OS; however, some reports have used CD68 as a pan TAM marker [69]. Some studies have also used CD80 and CD86 as M1-TAM markers for OS [36]; however, the use of M1-TAM markers in OS remains controversial.

#### 3.1.5. CD163

CD163 was discovered as a receptor that removes hemoglobin by mediating endocytosis of the haptoglobin-hemoglobin complex [70].

Several reports on CD163, a marker of tumor-promoting M2 TAMs in carcinomas, indicate that high levels of its expression correlate negatively with prognosis [71]. In prostate cancer, elevated PD-L1 expression was significantly correlated with CD163^+^ TAM invasion and Gleason score; furthermore, patients with high levels of CD163^+^ TAM and PD-L1 expression had shorter biochemical recurrence-free survival [72]. In CRC, CD163^+^ TAMs in the invasive front correlated with epithelial-mesenchymal transition (EMT), percentage of mesenchymal circulating tumor cells, and poor prognosis [73]. In prostate cancer, high expression of the circular RNA, circSMARCC1, correlated positively with colonization of CD68^+^/CD163^+^/CD206^+^ TAM colonization in the TME [74]. Expression of dynamin-related protein 1 (Drp1), the most important protein for mitochondrial division, showed a significant positive correlation with the percentage of CD163^+^ cells in hepatocellular carcinoma (HCC). A high Drp1 expression or patients with HCC with high Drp1 expression or high CD163^+^ TAM infiltration showed significantly lower overall and recurrence-free survival [75]. Patients with high CD25^+^ TIL/high CD163^+^ TAMs had a lower probability of recurrence-free lung cancer [76]. In cervical cancer, CD163^+^ M2-like macrophage infiltration correlated with increased PD-L1 expression in tumor cells, and PD-L1 expression was significantly correlated with shorter recurrence-free survival, and infiltration of moderate or higher CD163^+^ macrophages was also significantly correlated with recurrence-free survival [77]. Macrophage colony-stimulating factor (M-CSF), TGF-β, and vascular endothelial growth factor (VEGF) from primary tumor supernatants of breast cancer-induced healthy donor blood monocytes to differentiate into CD163-high CD86-low IL-10-high M2-like macrophages [78]. High expression of the glycogen branching enzyme (GBE1), a key gene involved in the regulation of glycogen metabolism in lung adenocarcinoma, correlated with decreased overall survival and advanced TNM classification [79]. In clear cell renal cell carcinoma (ccRCC), the tumor suppressor ring finger protein 43 (RNF43) negatively correlated with the level of CD163+ TAM invasion, and combining these with the TNM stage could significantly enhance the veracity in forecasting ccRCC postoperative outcomes [80]. However, iNOS and CD163^+^ macrophage infiltration have been reported to correlate with improved prognosis in CRC [81]. In sarcomas, a high number of CD163^+^ TAMs is associated with poor prognosis [52], and IHC evaluation of CD163^+^ macrophage infiltration in high-grade leiomyosarcoma, liposarcoma, and synovial sarcoma showed that higher CD163^+^ macrophage infiltration was observed in high-grade liposarcoma compared to low-grade liposarcoma and was associated with shorter metastasis-free survival [82]. Some reports suggest that CD163^+^ TAMs enhance T-cell depletion in OS [40], and high CD163 expression correlates with poor OS prognosis [41]. In contrast, other studies suggest that high CD163 levels are independently and significantly associated with improved overall survival and longer metastasis-free survival in OS [42].

#### 3.1.6. CD204 (MSR1)

CD204 is a prototypic member of the transmembrane receptor family called scavenger receptors and is preferentially expressed on myeloid cells such as macrophages and dendritic cells [83]. The Cancer Genome Atlas (TCGA) database showed that CD204 expression increases in various subtypes of breast cancer. Furthermore, the GEO dataset shows that high CD204 expression is associated with poor clinical outcomes, and CD204^+^ TAMs promote breast cancer cell migration and invasion [84]. The pathological study indicated that increased density of CD204+ TAMs was closely associated with cancer cell proliferation and worse clinical course in breast cancer, and the density of CD204+ TAMs was a significant prognostic factor rather than the density of CD68+ or CD163+ TAMs [85]. There is a negative relationship between high CD204^+^ TAM infiltration and both overall and progression-free survival in ccRCC, as tissues with high CD204 expression have higher PD1^+^ LAG3^+^ CD8^+^ T-cell infiltration than those with low expression [86]. Non-small cell lung cancer (NSCLC), bladder cancer with muscle layer invasion, and upper urinary tract cancer were associated with higher numbers of CD204^+^ TAMs and shorter survival [87,88,89].

In OS, M2 TAMs induced by IL-4 and IL-13 increase CD204 expression and activate the NF-κB/miR-181α-5p/RASF1A/Wnt pathway by increasing IL-1β production, which promotes OS cell progression and metastasis [43]. Stimulation of CD14^+^ PBMCs with M-CSF and 50% OS conditioned medium increased the expression of macrophage-associated molecules CD68, CD163, CD204, IL-10, and CCL1 [44]. In contrast, there are contradictory reports that CD204 has significant effects on anti-metastasis in OS, which may prolong patients’ overall survival [45].

#### 3.1.7. CD206 or C-Type Mannose Receptor 1 (MRC1)

CD206 (MRC1) is a 175 kDa type I transmembrane glycoprotein that is expressed on a variety of tissue-resident macrophages and is known as the M2 macrophage marker [32].

A systematic review found that high-density CD206 TAMs were associated with poor overall survival in hepatocellular carcinoma [90]. In OS, macrophages activated by mifamurtide show increased levels of the M1 and M2 markers iNOS and CD206, respectively, and increased gene expression of the inflammatory cytokines IL-1β and IL-6 and the anti-inflammatory cytokines IL-4 and IL-10. Mifamurtide switches macrophage polarization to a TAM-like intermediate M1/M2 phenotype and reduces IL-17R levels and STAT3 activation, thereby inhibiting cell growth and inducing tumor-cell differentiation [46].

#### 3.1.8. Dendritic Cell-Specific C-Type Lectin (DC-SIGN) or CD209

DC-SIGN, also called CD209, was initially identified as a DC surface protein that binds to the HIV-1 envelope glycoprotein gp120 [91].

High levels of CD209^+^ TAM infiltration in muscle layer invasive bladder cancer were associated with CD8^+^ T-cell tolerance, poor prognosis, and non-response to adjuvant chemotherapy [92]. CD209^+^ M2 TAMs infiltrate OS and promote OS progression by activating cancer stem cells [47]. CD209 was suggested as a marker for tissue-resident macrophages [93] and might be useful to distinguish be-tween monocyte-derived TAMs and tissue-resident TAMs.

### 3.2. Are M1/M2 TAM Markers Reliable Indicators of OS Prognosis?

In summary, M1/M2 markers in OS differ from anti/pro-tumor markers in carcinomas. TAMs express multiple markers, and there may be unknown markers. Interestingly, some studies show a poor prognosis, for example, CD163, while others show a good prognosis with the same marker. In OS, single-cell analysis showed that TAMs are not significantly polarized M1/M2 in lung metastases [94], and gene set variation analysis (GSVA) analysis showed the presence of M2-TAMs with relatively high expression levels of the M1-TAM marker gene in primary [10]. Some markers that are supposed to be expressed in either one or other polarity are known to be expressed in both M1 and M2. These contradictory marker reports make it difficult to pinpoint a particular marker for therapy with reasonable clarity [95,96]. Using M1/M2 markers to divide TAM into anti/pro-tumor is unreliable and should be considered. Therefore, further research on TAM markers in OS is needed in the future.

## 4. TAM-Related Biomarkers in the TME

**Figure 2:**
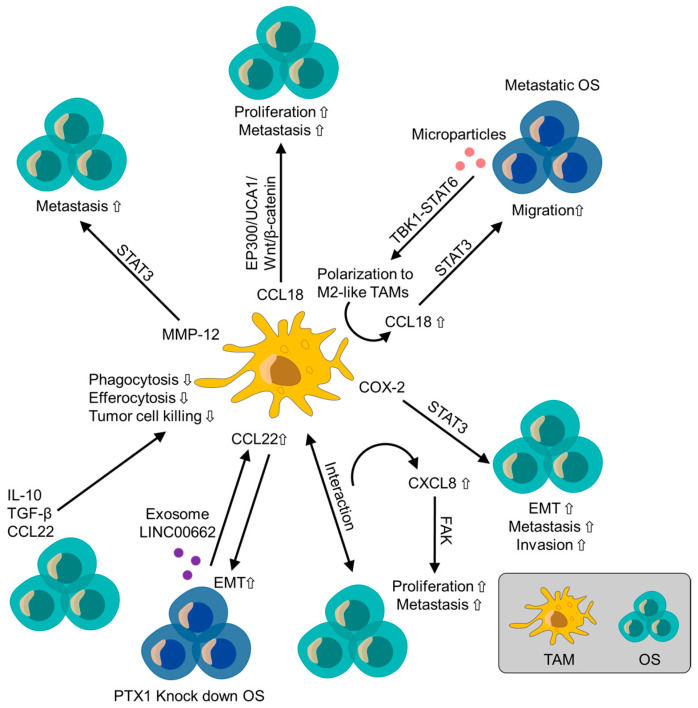
Biomarkers related to osteosarcoma and tumor-associated macrophage. COX-2: Cyclooxygenase-2, EMT; Epithelial-mesenchymal transition, OS: Osteosarcoma, PITX1: Paired-like homeodomain transcription factor 1, TAM: Tumor-associated macrophage.

Chemokines and cytokines produced by TAMs have attracted much attention as targets for therapy. Chemokines comprise four subfamilies, including CCL, CXCL, CX3C, and XCL [97]. Cytokines include TNFs, ILs, phosphokines, monokines, IFNs, and colony-stimulating factors. Here, we discuss the TAM-related biomarkers that have been identified in OS (Figure 2).

### 4.1. Chemokines

#### 4.1.1. CCL2/Monocyte Chemoattractant Protein-1 (MCP-1)

TAM recruitment into tumor tissue can be inhibited by blocking monocyte chemotactic chemokines and cytokines or their receptors. CCL2 (MCP-1) binds to G protein-coupled receptors and plays a major role in promoting inflammation by modulating the activity of monocytes and basophils [98].

Monocyte recruitment to tumor metastatic sites occurs in a CCL2-dependent manner, and the conversion of monocytes to macrophages promotes tumor growth, metastatic tumor survival, proliferation, and poor prognosis in various types of cancers such as breast and small cell lung cancers, and glioblastoma multiforme [99,100,101]. In breast cancer, blockade of the CCL2/CCR2 axis reduces macrophage infiltration and decreases tumor growth [102]. In inflammatory breast cancer, knockdown of CCL2 markedly reduces macrophage density, tumor growth, skin erythema, and metastasis [103]. Overexpression of Drp1 in HCC increases CCL2 gene expression and protein production in HCC cells, as well as CD163^+^ TAM infiltration [75]. Combining paclitaxel and carboplatin with anti-CCL2 antibody enhances the therapeutic effect in a mouse model of ovarian cancer [104].

In OS, CCL2 also promotes MMP-9 expression, cell migration, and cell invasion via CCR2, c-Raf, MAPK, and AP-1 signaling [105]. Plasma levels of MCP-1 strongly correlate with OS progression and osteolysis, and administration of zoledronic acid directly attenuates OS RANKL/CCL2 production and reduces tumor-induced bone destruction [106]. Inhibition of the CCL2/CCR2 axis by bindarit, a CCL2 inhibitor, blocks the inhibitory effect of the tumor suppressor TIPE1 on OS growth [107].

#### 4.1.2. CCL5 (RANTES)

CCL5 is a chemokine expressed by inflammatory cells such as T cells and monocytes/macrophages [108]. TAM-derived CCL5 promotes self-renewal of prostate cancer stem cells (PCSCs) and prostate cancer metastasis via activation of β-catenin/STAT3 signaling [109]. Cytotoxic stress from infiltrating lymphocytes stimulates breast cancer cells to produce CCL5, which activates CCR5 signaling and mobilizes TAMs, and TAM-derived factors (OPN, HB-EGF, and IL-6) stimulate breast cancer cell growth [110]. A positive feedback loop of CCL5-CCR5 and CCL18-PIPTNM3 between myofibroblasts and TAMs is established in malignant phyllodes tumors (PT) and promotes their malignant progression. Furthermore, targeting CCR5 using the CCR5-inhibitor maraviroc is a new strategy to treat malignant PT [111]. FROUNT is an activation cofactor of CCR2 and CCR5 involved in macrophage chemotaxis, and a FROUNT inhibitor enhances the anticancer effect of immunotherapy via suppressing TAM infiltration in cancer tissue [112]. A FROUNT inhibitor was also shown to suppress TAM invasion and cancer growth in breast cancer models [110]. CCL5 produced by TAMs in prostate cancer enhances resistance to PTX and DOX via the STAT3/Nanog signaling pathway [113].

In OS, the exogenous CCL5-CCR5 axis induces cell migration via the MEK/ERK signaling pathway or promotes VEGF expression and angiogenesis via the PKCδ/c-Src signaling pathway [114,115].

#### 4.1.3. CCL18

CCL18 is secreted mainly by cells of the bone-associated system and produced by M2 macrophages induced by IL-4 and IL-13 [116]. It is secreted by TAMs and promotes tumor-cell proliferation via the JAK2/STAT3 signaling pathway. CCL18 blockade can significantly prevent esophageal squamous cell carcinoma progression [117]. TAM-derived CCL18 induces EMT in breast cancer and activates ERK and Akt/GSK-3β/Snail signaling in human umbilical vein endothelial cells, thereby contributing to its pro-angiogenic effect [118]. TAM-derived CCL18-PITPNM3 axis promotes breast cancer metastasis [119].

Macrophage-derived CCL18 promotes OS proliferation and metastasis via the EP300/UCA1/Wnt/β-catenin pathway [120]. OS tumor-derived microparticles promote the polarization of macrophages to an M2-like phenotype through TBK1-STAT6 signaling and mediate migration of OS cells through CCL18/STAT3 signaling [121].

#### 4.1.4. CCL22

TAM-derived CCL22 promotes EMT and metastasis via the DGKα/FAK signaling pathway in metastatic esophageal squamous cell carcinoma [122]. CCR4^+^ head and neck squamous cell carcinoma (HNSCC), compared to CCR4-, promotes lymph node metastasis with TAM-derived CCL22 [123].

Stimulation of macrophages with exosomes from metastatic OS cell lines K7M3 and DLM8 increased the expression levels of IL-10, TGF-β2, and CCL22; however, their expression levels did not increase in non-metastatic cell lines K7 and DUNN [124]. Paired-like homeodomain transcription factor 1 (PITX1) knockdown OS cell line-derived exosome LINC00662 causes OS EMT via CCL22 production in M2 TAMs [125].

#### 4.1.5. CXCL1

CXCL1 is a secreted growth factor that signals through the G-protein coupled receptor, CXCR 2. This protein is involved in inflammation and functions as a chemoattractant for neutrophils [126].

TAMs and cancer-associated fibroblasts (CAFs) in the TME of bladder cancer are associated with tumor invasion, recurrence, disease progression, and drug resistance through CXCL1 [127]. In triple-negative breast cancer (TNBC), M2 macrophages highly express CXCL1 and enhance PD-L1 expression in TNBC cells [128].

In OS, CXCL1, by paracrine means, promotes the migration and invasion of OS cells into the lung via the CXCR2/FAK/PI3K/Akt pathway, or CXCL1 from pulmonary vessels, promotes OS migration [129,130].

#### 4.1.6. CXCL5

CXCL5 is also known as the neutrophil-activating peptide, ENA-78. It is an inflammatory chemokine produced simultaneously with CXCL8 in response to stimulation by IL-1 or TNF-α [131]. TAM-derived CXCL5 promotes chemotherapy resistance in gastric cancer via the PI3K/AKT/mTOR pathway [132]. Although the effects of TAM-derived CXCL5 with respect to OS are unknown, there are reports that OS cells show enhanced migration and invasion when using exogenous CXCL5 or a titrated medium of hFoB1.19 cells overexpressing CXCL5 [133].

#### 4.1.7. CXCL6/Granulocyte Chemotaxis Protein 2 (GCP-2)

CXCL6 is a small cytokine of the CXC chemokine family and a chemoattractant of neutrophilic granulocytes [134]. In hepatocellular carcinoma, there is a positive correlation between CXCL6 and CD163 expression in patient samples, and TAMs promote cancer cell proliferation and migration by activating CXCR2/IFN-g/p38 MAPK/NF-κB signaling [135].

Exogenous CXCL6 promotes cell proliferation, migration, invasion, and EMT via activation of the PI3K/AKT and Wnt/β-catenin pathways in OS [136].

#### 4.1.8. CXCL8 (IL-8)

CXCL8 (IL-8) is a chemokine that binds primarily to G protein-coupled receptors, CXCR1 and CXCR2, and induces inflammation via neutrophils and granulocytes [137,138].

TAM-derived CXCL8 promotes MMP-9, VEGF, and E-cadherin expression in bladder cancer cells and enhances bladder cancer cell migration, invasion, and angiogenesis [139]. TAMs also promote papillary thyroid carcinoma invasion via CXCL8 secretion [140]. Hypoxia promotes gastric cancer progression by activating the release of CXCL8 by macrophages and the CXCL8/CXCR1/2-JAK/STAT1 pathway. This pathway directly promotes the expression of gastric cancer-derived IL-10 and forms a positive feedback loop that further releases macrophage-derived CXCL8 under the IL-10/NF-κB/CXCL8 axis [141]. Macrophages stimulated with a conditioned medium of esophageal squamous cell carcinoma cell lines showed increased expression of CXCL8. Recombinant human CXCL8 induced migration and invasion of esophageal squamous cell carcinoma cell lines by phosphorylation of Akt and Erk1/2. In patient samples, high expression levels of CXCL8 in esophageal squamous cell carcinoma tissue were significantly associated with lymph node metastasis and poor prognosis [142]. TAM-derived CXCL8 promotes breast cancer stem cell (BCSC) self-renewal and elevates breast cancer metastasis, and the CXCR2 antagonist danirixin inhibits the TAM/CXCL8 regulatory mechanism to eliminate BCSCs [143].

In OS, CXCL8 enhances invasion and inhibits late apoptosis of human OS cell lines in vitro by affecting the PI3K/Akt signaling pathway and upregulating MMP expression [144]. Interaction between OS and macrophages results in increased CXCL8 production in both. Increased CXCL8 promotes OS proliferation and metastasis via FAK phosphorylation of the OS cells [34].

### 4.2. Cytokines and Enzymes

#### 4.2.1. IL-6

IL-6 was discovered in 1986 as a B-cell differentiation factor that differentiates activated B cells into immunoglobulin-producing cells [145]. There are several reports on the role of IL-6 in the TME of carcinomas.

M2-like macrophages drive glioma, angiogenic mimicry by amplifying IL-6 secretion in glioma cells via the PKC pathway [146]. TAM-derived IL-6 activated the JAK2/STAT3 pathway, and activated STAT3 inhibited transcription of the tumor suppressor miR-506-3p in CRC cells. miR-506-3p, a key miRNA-regulating FoxQ1, was downregulated in CRC cells, resulting in increased FoxQ1 expression and consequent production of CCL2, which promotes macrophage mobilization [73].

Although not a TAM in OS, rIL-6 and mesenchymal stem cell-derived IL-6 have been reported to be associated with OS. Exogenous IL-6 and IL-6R interaction increases intercellular adhesion molecule-1 (ICAM-1) expression via the ILK/Akt/c-Jun/AP-1-dependent pathway and induces migration of human OS cells [147]. Mesenchymal stem cells activate STAT3 in OS cells through secretion of IL-6, thereby increasing tumor growth and metastasis and decreasing apoptosis [148].

#### 4.2.2. Cyclooxygenase-2 (COX-2)

COX-2 is an important rate-limiting enzyme that catalyzes the conversion of arachidonic acid to various prostaglandins, including prostaglandin E2 (PGE2) [149,150].

Endocrine-resistant breast cancer cells promote COX-2 expression in TAMs via the JNK/c-Myc/arginase-1 pathway and further promote endocrine resistance in breast cancer cells by activating the PI3K/Akt/mTOR pathway, forming a positive feedback loop between TAMs and breast cancer cells [150]. Post-irradiation TAMs express higher levels of COX-2 and promote early tumor growth of prostate cancer in vivo [151].

TAMs promote OS metastasis and invasion by activating the COX-2/STAT3 axis and EMT; celecoxib suppresses this promoting effect of TAMs [152].

#### 4.2.3. Matrix Metalloproteinase-12 (MMP-12)

MMPs are a family of over 20 zinc-dependent endopeptidases, first introduced as secreted proteases capable of cleaving extracellular matrix proteins. They play a major role in cell differentiation, proliferation, wound healing, apoptosis, and angiogenesis [153].

Among them, MMP-12 is mainly secreted by macrophages and degrades a variety of extracellular matrix proteins, including elastin (abundant in ligaments, lungs, arteries, etc.) [154]. Elevated levels of MMP-12 derived from tumor-infiltrating macrophages were associated with shorter overall survival in plasma from patients with muscle-invasive uroepithelial bladder cancer [155].

MMP-12, a TAM-secreted elastase, promotes OS metastasis, and all-trans retinoic acid (ATRA) suppresses MMP-12 production [156].

In addition, in esophageal squamous cell carcinoma, TAM-derived CCL1 promotes tumor growth through the Akt/proline-rich Akt substrate of 40 kDa/mammalian target of rapamycin (mTOR) pathway [157], and in prostate, ovarian, and renal cancers. It has been reported that CCL20 promotes tumor growth in cell cancers [74,158,159].

It is possible that TAM-related biomarkers, such as those reported in carcinomas, may be associated with growth and metastasis in OS. Studies on TAM-producing biomarkers in OS are progressing gradually but remain insufficient. Future research in this field is expected to yield favorable results.

## 5. Origin of TAMs; Aspect from Single-Cell RNA-Sequence Analysis

**Figure 3:**
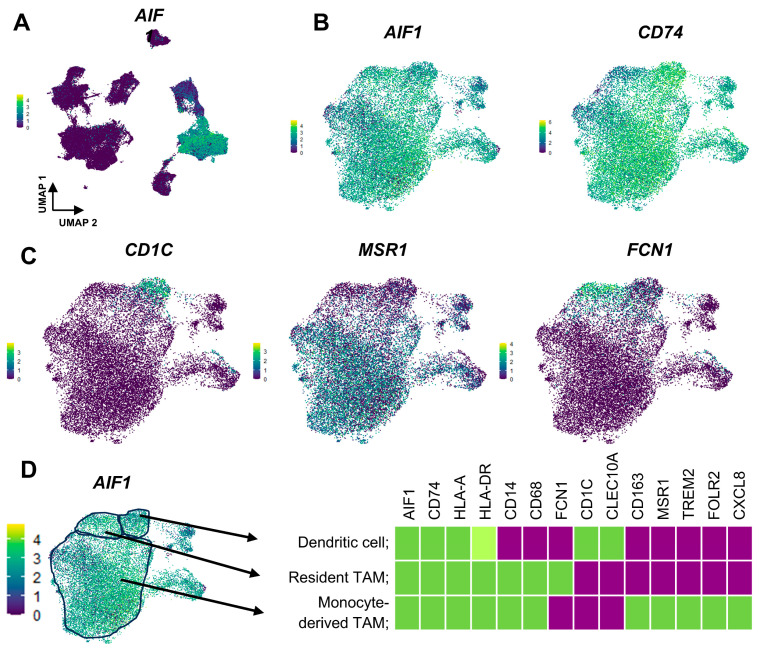
Myeloid cell subgroups suggested by single cell-RNA sequence. (**A**); Detailed methods and results were described and shown in Supplementary Materials UMAP plot of *AIF1* (*Iba-1*) gene expression across all the cells (GSE152048) is presented. (**B**); UMAP plot of the selected *AIF1 (Iba-1)* gene and *CD74* gene are presented. (**C**); UMAP plot of selected genes indicated the myeloid cells were composed of their subpopulations (dendritic cells, resident TAMs, and monocyte-derived TAMs). (**D**); Gene expression scheme in each subpopulation was shown.

Single-cell RNA-sequence analysis was performed using a published data set (GSE152048) as described in Supplementary Figures S1–S3. *AIF1(Iba1)* gene expression was suggested as the most suitable marker for myeloid lineage cells (Figure 3A), and myeloid cells also strongly expressed *CD74* (Figure 3B). Myeloid cells were suggested to be divided broadly into three groups. *CD1c*, *MSR1*, and *FCN1* expressing cells seemed to be dendritic cells, monocyte-derived TAMs, and tissue-resident TAMs, respectively (Figure 3C). Myeloid cells were all strongly expressed HLA-class I and HLA-class II genes, and the strongest HLA-class II expression was seen in dendritic cells (Figure 3D). TAMs are mainly composed of monocyte-derived TAMs, which express protumor molecules such as CD163, MSR1(CD204), TREM2, FOLR2, and CXCL8 genes. These indicated that protumor TAMs predominantly originated from circulating monocytes, and blocking the chemotaxis of monocytes into tumor tissue would be suitable for the reduction of protumor TAMs.

## 6. Conclusions 

Several unknowns remain regarding the TME, or TAM, in OS. TAMs are highly valuable therapeutic targets, as reported in other carcinomas, and this may be true in OS as well. M1-like TAMs secrete NO, which induces Th1 and can directly suppress cancer cells. Activated Th1 responses also promote the activation of M1-like TAMs, CD8+ T cells, IgG B cells, and IFN-γ-producing CD4+ T cells, exerting inflammatory and anti-tumor effects [160]. Therefore, a therapeutic strategy targeting TAMs is to repolarize M2-like to M1-like TAMs. In colorectal cancer, regulation of the Agpat4/LPA/p38/p65 axis has been reported to control repolarization to M1-like TAMs, T cell activity, and tumor progression [161]. In osteosarcoma, ATRA, asiaticoside, photothermal therapy, mifamurtide, and others have been reported to repolarize M2-like to M1-like TAMs [162]. There are also reports that chemokines/cytokines from TAMs are elevated by anticancer drugs and associated with increased resistance and decreased sensitivity to anticancer drugs [163,164,165].

Therapeutic targeting of TAM markers and related biomarkers in OS, in combination with currently available OS anticancer therapy, could potentially reduce the side effects of anticancer drugs and improve treatment outcomes. This review summarizes TAM markers and TAM-related biomarkers. A better understanding of OS and TAMs in the TME has great potential to break the current status quo of OS therapy, which has stalled in improving prognosis.

## Figures and Tables

**Table 1:** Macrophage markers in osteosarcoma (OS).

TAM Marker	Function	Outcome	References
Iba-1	Pan TAM marker	Iba-1^+^ TAM primary site < metastases	[34]
iNOS	Anti-tumor	Negative correlation between expression and lung metastasis. Predictor of non-metastasis	[35]
CD80	Anti-tumor	CD80^+^ TAM primary site > metastases Decreased M1 TAM is associated with osteosarcoma metastasis	[36]
CD86	Anti-tumor	Anti-PD-1 treatment causes repolarization from M2 to M1 TAMs and suppresses pulmonary metastasis of osteosarcoma	[37]
CD86	Anti-tumor	Positive correlation between expression and prognosis Mainly expressed in M2 TAMs	[38]
CD68	Anti-tumor	Positive correlation between CD68^+^ TAM infiltration and prognosis	[39]
CD68	-	The amount of CD68^+^ TAM is the same with and without metastasis	[35]
CD163	Pro-tumor	Enhances T-cell suppression	[40]
CD163	Pro-tumor	High CD163 expression correlates with poor OS prognosis	[41]
CD163	Anti-tumor	Positive correlation with improved overall survival and prolonged metastasis-free survival	[42]
CD204	Pro-tumor	Promotes OS cell progression and metastasis	[43]
CD68 CD163 CD204	Pro-tumor	TAMs prepared by stimulating CD14^+^ PBMCs with M-CSF and 50% OS conditioned medium promote migration of OS cells	[44]
CD204	Anti-tumor	Positive correlation of expression with resistance to metastasis and prolonged overall survival	[45]
iNOS CD206	-	Macrophages activated by mifamurtide have increased levels of both iNOS and CD206, switch to an M1/M2 phenotype, inhibit cell proliferation, and induce tumor cell differentiation	[46]
CD209	Pro-tumor	Promotes OS progression by activating cancer stem cells	[47]

M-CSF: Macrophage colony-stimulating factor, PBMC: Peripheral blood mononuclear cells, PD-1: Programmed cell death 1, TAM: Tumor-associated macrophage.

## Supplementary Materials

The following supporting information can be downloaded at https://www.mdpi.com/article/10.3390/cancers16162801/s1, Figure S1: (a) UMAP plot of the cells (n = 93,357) from 11 osteosarcoma tissues (GSE152048, primary, n = 7, recurrent, n = 2, and lung metastatic, n = 2) clustered into 34 clusters. (b) UMAP plot of the expression of gene *AIF1 (Iba-1)* across all the cells. (c) Violin plot of the expression of gene *AIF1 (Iba-1)* across all 34 clusters. (d) UMAP plot of the selected *AIF1 (Iba-1)* positive cells (included cluster 0, 11, 17, 22, 33, and part of 13 which is positive for *AIF1*). The *AIF1(Iba-1)* positive cells serve as myeloid lineage cells and re-clustered into 13 clusters; Figure S2: UMAP plots of the expression of the indicated genes in the *AIF1(Iba-1)* positive cells of all 11 osteosarcoma tissues; Figure S3: Violin plots of the expression of the indicated genes in the *AIF1(Iba-1)* positive cells across primary and metastatic/recurrent tissues.

## References

[1] Allison D.C., Carney S.C., Ahlmann E.R., Hendifar A., Chawla S., Fedenko A., Angeles C., Menendez L.R. (2012). A Meta-analysis of osteosarcoma outcomes in the modern medical era. Sarcoma.

[2] Isakoff M.S., Bielack S.S., Meltzer P., Gorlick R. (2015). Osteosarcoma: Current Treatment and a Collaborative Pathway to Success. J. Clin. Oncol..

[3] Meazza C., Scanagatta P. (2016). Metastatic osteosarcoma: A challenging multidisciplinary treatment. Expert Rev. Anticancer. Ther..

[4] Smeland S., Bruland Ø.S., Hjorth L., Brosjö O., Bjerkehagen B., Österlundh G., Jakobson Å., Hall K.S., Monge O.R., Björk O. (2011). Results of the Scandinavian Sarcoma Group XIV protocol for classical osteosarcoma: 63 patients with a minimum follow-up of 4 years. Acta Orthop..

[5] Inagaki Y., Hookway E., Williams K.A., Hassan A.B., Oppermann U., Tanaka Y., Soilleux E., Athanasou N.A. (2016). Dendritic and mast cell involvement in the inflammatory response to primary malignant bone tumours. Clin. Sarcoma Res..

[6] Wang F., Yang L., Gao Q., Huang L., Wang L., Wang J., Wang S., Zhang B., Zhang Y. (2015). CD163+CD14+ macrophages, a potential immune biomarker for malignant pleural effusion. Cancer Immunol. Immunother..

[7] Tang X. (2013). Tumor-associated macrophages as potential diagnostic and prognostic biomarkers in breast cancer. Cancer Lett..

[8] Kim K.-J., Wen X.-Y., Yang H.K., Kim W.H., Kang G.H. (2015). Prognostic Implication of M2 Macrophages Are Determined by the Proportional Balance of Tumor Associated Macrophages and Tumor Infiltrating Lymphocytes in Microsatellite-Unstable Gastric Carcinoma. PLoS ONE.

[9] Shigeoka M., Urakawa N., Nakamura T., Nishio M., Watajima T., Kuroda D., Komori T., Kakeji Y., Semba S., Yokozaki H. (2013). Tumor associated macrophage expressing CD204 is associated with tumor aggressiveness of esophageal squamous cell carcinoma. Cancer Sci..

[10] Zhou Y., Yang D., Yang Q., Lv X., Huang W., Zhou Z., Wang Y., Zhang Z., Yuan T., Ding X. (2020). Single-cell RNA landscape of intratumoral heterogeneity and immunosuppressive microenvironment in advanced osteosarcoma. Nat. Commun..

[11] Ichikawa J., Ando T., Kawasaki T., Sasaki T., Shirai T., Tsukiji N., Kimura Y., Aoki K., Hayakawa K., Suzuki-Inoue K. (2020). Role of Platelet C-Type Lectin-Like Receptor 2 in Promoting Lung Metastasis in Osteosarcoma. J. Bone Miner. Res..

[12] Saito M., Ichikawa J., Ando T., Schoenecker J.G., Ohba T., Koyama K., Suzuki-Inoue K., Haro H. (2018). Platelet-Derived TGF-β Induces Tissue Factor Expression via the Smad3 Pathway in Osteosarcoma Cells. J. Bone Miner. Res..

[13] Tan S., Chao R. (2023). An Exploration of Osteosarcoma Metastasis Diagnostic Markers Based on Tumor-Associated Neutrophils. Discov. Med..

[14] Du L., Han X.-G., Tu B., Wang M.-Q., Qiao H., Zhang S.-H., Fan Q.-M., Tang T.-T. (2018). CXCR1/Akt signaling activation induced by mesenchymal stem cell-derived IL-8 promotes osteosarcoma cell anoikis resistance and pulmonary metastasis. Cell Death Dis..

[15] Kawano M., Tanaka K., Itonaga I., Iwasaki T., Tsumura H. (2018). Interaction between human osteosarcoma and mesenchymal stem cells via an interleukin-8 signaling loop in the tumor microenvironment. Cell Commun. Signal..

[16] Tsukamoto H., Komohara Y., Oshiumi H. (2021). The role of macrophages in anti-tumor immune responses: Pathological significance and potential as therapeutic targets. Hum. Cell.

[17] Chen J.J., Yao P.-L., Yuan A., Hong T.-M., Shun C.-T., Kuo M.-L., Lee Y.-C., Yang P.-C. (2003). Up-regulation of tumor interleukin-8 expression by infiltrating macrophages: Its correlation with tumor angiogenesis and patient survival in non-small cell lung cancer. Clin. Cancer Res..

[18] Stein M., Keshav S., Harris N., Gordon S. (1992). Interleukin 4 potently enhances murine macrophage mannose receptor activity: A marker of alternative immunologic macrophage activation. J. Exp. Med..

[19] Mills C.D., Kincaid K., Alt J.M., Heilman M.J., Hill A.M. (2000). M-1/M-2 Macrophages and the Th1/Th2 Paradigm. J. Immunol..

[20] Martinez F.O., Gordon S. (2014). The M1 and M2 paradigm of macrophage activation: Time for reassessment. F1000Prime Rep..

[21] Heideveld E., Hampton-O’Neil L.A., Cross S.J., van Alphen F.P.J., van den Biggelaar M., Toye A.M., van den Akker E. (2018). Glucocorticoids induce differentiation of monocytes towards macrophages that share functional and phenotypical aspects with erythroblastic island macrophages. Haematologica.

[22] Shapouri-Moghaddam A., Mohammadian S., Vazini H., Taghadosi M., Esmaeili S.-A., Mardani F., Seifi B., Mohammadi A., Afshari J.T., Sahebkar A. (2018). Macrophage plasticity, polarization, and function in health and disease. J. Cell. Physiol..

[23] Gordon S. (2003). Alternative activation of macrophages. Nat. Rev. Immunol..

[24] Takeya M., Komohara Y. (2016). Role of tumor-associated macrophages in human malignancies: Friend or foe?. Pathol. Int..

[25] Qian B.-Z., Pollard J.W. (2010). Macrophage diversity enhances tumor progression and metastasis. Cell.

[26] Wynn T.A., Chawla A., Pollard J.W. (2013). Macrophage biology in development, homeostasis and disease. Nature.

[27] Shinchi Y., Ishizuka S., Komohara Y., Matsubara E., Mito R., Pan C., Yoshii D., Yonemitsu K., Fujiwara Y., Ikeda K. (2022). The expression of PD-1 ligand 1 on macrophages and its clinical impacts and mechanisms in lung adenocarcinoma. Cancer Immunol. Immunother..

[28] Barros M.H.M., Hauck F., Dreyer J.H., Kempkes B., Niedobitek G. (2013). Macrophage polarisation: An immunohistochemical approach for identifying M1 and M2 macrophages. PLoS ONE.

[29] Ambarus C., Krausz S., van Eijk M., Hamann J., Radstake T., Reedquist K., Tak P., Baeten D. (2012). Systematic validation of specific phenotypic markers for in vitro polarized human macrophages. J. Immunol. Methods.

[30] Stöger J.L., Gijbels M.J., van der Velden S., Manca M., van der Loos C.M., Biessen E.A., Daemen M.J., Lutgens E., de Winther M.P. (2012). Distribution of macrophage polarization markers in human atherosclerosis. Atherosclerosis.

[31] Ohri C.M., Shikotra A., Green R.H., Waller D.A., Bradding P. (2009). Macrophages within NSCLC tumour islets are predominantly of a cytotoxic M1 phenotype associated with extended survival. Eur. Respir. J..

[32] Rőszer T. (2015). Understanding the Mysterious M2 Macrophage through Activation Markers and Effector Mechanisms. Mediat. Inflamm..

[33] Durafourt B.A., Moore C.S., Zammit D.A., Johnson T.A., Zaguia F., Guiot M., Bar-Or A., Antel J.P. (2012). Comparison of polarization properties of human adult microglia and blood-derived macrophages. Glia.

[34] Tatsuno R., Ichikawa J., Komohara Y., Pan C., Kawasaki T., Enomoto A., Aoki K., Hayakawa K., Iwata S., Jubashi T. (2024). Pivotal role of IL-8 derived from the interaction between osteosarcoma and tumor-associated macrophages in osteosarcoma growth and metastasis via the FAK pathway. Cell Death Dis..

[35] Dumars C., Ngyuen J.-M., Gaultier A., Lanel R., Corradini N., Gouin F., Heymann D., Heymann M.-F. (2016). Dysregulation of macrophage polarization is associated with the metastatic process in osteosarcoma. Oncotarget.

[36] Wang Z., Wu H., Chen Y., Chen H., Yuan W., Wang X. (2021). The Heterogeneity of Infiltrating Macrophages in Metastatic Osteosarcoma and Its Correlation with Immunotherapy. J. Oncol..

[37] Dhupkar P., Gordon N., Stewart J., Kleinerman E.S. (2018). Anti-PD-1 therapy redirects macrophages from an M2 to an M1 phenotype inducing regression of OS lung metastases. Cancer Med..

[38] Li J., Su L., Xiao X., Wu F., Du G., Guo X., Kong F., Yao J., Zhu H. (2022). Development and Validation of Novel Prognostic Models for Immune-Related Genes in Osteosarcoma. Front. Mol. Biosci..

[39] Song Y.-J., Xu Y., Zhu X., Fu J., Deng C., Chen H., Xu H., Song G., Lu J., Tang Q. (2020). Immune Landscape of the Tumor Microenvironment Identifies Prognostic Gene Signature CD4/CD68/CSF1R in Osteosarcoma. Front. Oncol..

[40] Han Q., Shi H., Liu F. (2016). CD163 + M2-type tumor-associated macrophage support the suppression of tumor-infiltrating T cells in osteosarcoma. Int. Immunopharmacol..

[41] Zheng S., Peng J., Jia J., Wu T., Cheng X. (2021). Revealing the link between macrophage in microenvironment of osteosarcoma and poor prognosis by utilizing the Integrated analysis. J. Musculoskelet. Neuronal Interact..

[42] Gomez-Brouchet A., Illac C., Gilhodes J., Bouvier C., Aubert S., Guinebretiere J.-M., Marie B., Larousserie F., Entz-Werlé N., de Pinieux G. (2017). CD163-positive tumor-associated macrophages and CD8-positive cytotoxic lymphocytes are powerful diagnostic markers for the therapeutic stratification of osteosarcoma patients: An immunohistochemical analysis of the biopsies fromthe French OS2006 phase 3 trial. OncoImmunology.

[43] Han Z.-P., Liu D.-B., Wu L.-Q., Li Q., Wang Z.-G., Zang X.-F. (2020). IL-1β secreted by macrophage M2 promotes metastasis of osteosarcoma via NF-κB/miR-181α-5p/RASSF1A/Wnt pathway. Transl. Cancer Res..

[44] He F., Ding G., Jiang W., Fan X., Zhu L. (2021). Effect of tumor-associated macrophages on lncRNA PURPL/miR-363/PDZD2 axis in osteosarcoma cells. Cell Death Discov..

[45] Chen Z., Huang H., Wang Y., Zhan F., Quan Z. (2020). Identification of Immune-Related Genes MSR1 and TLR7 in Relation to Macrophage and Type-2 T-Helper Cells in Osteosarcoma Tumor Micro-Environments as Anti-metastasis Signatures. Front. Mol. Biosci..

[46] Punzo F., Bellini G., Tortora C., Di Pinto D., Argenziano M., Pota E., Di Paola A., Di Martino M., Rossi F. (2020). Mifamurtide and TAM-like macrophages: Effect on proliferation, migration and differentiation of osteosarcoma cells. Oncotarget.

[47] Shao X.-J., Xiang S.-F., Chen Y.-Q., Zhang N., Cao J., Zhu H., Yang B., Zhou Q., Ying M.-D., He Q.-J. (2019). Inhibition of M2-like macrophages by all-trans retinoic acid prevents cancer initiation and stemness in osteosarcoma cells. Acta Pharmacol. Sin..

[48] Motoshima T., Miura Y., Wakigami N., Kusada N., Takano T., Inoshita N., Okaneya T., Sugiyama Y., Kamba T., Takeya M. (2018). Phenotypical change of tumor-associated macrophages in metastatic lesions of clear cell renal cell carcinoma. Med. Mol. Morphol..

[49] Yonemitsu K., Pan C., Fujiwara Y., Miyasato Y., Shiota T., Yano H., Hosaka S., Tamada K., Yamamoto Y., Komohara Y. (2022). GM-CSF derived from the inflammatory microenvironment potentially enhanced PD-L1 expression on tumor-associated macrophages in human breast cancer. Sci. Rep..

[50] Tartey S., Neale G., Vogel P., Malireddi R.S., Kanneganti T.-D. (2021). A MyD88/IL1R Axis Regulates PD-1 Expression on Tumor-Associated Macrophages and Sustains Their Immunosuppressive Function in Melanoma. Cancer Res..

[51] Komohara Y., Takeya H., Wakigami N., Kusada N., Bekki H., Ishihara S., Takeya M., Nakashima Y., Oda Y. (2019). Positive correlation between the density of macrophages and T-cells in undifferentiated sarcoma. Med. Mol. Morphol..

[52] Shiraishi D., Fujiwara Y., Horlad H., Saito Y., Iriki T., Tsuboki J., Cheng P., Nakagata N., Mizuta H., Bekki H. (2018). CD163 Is Required for Protumoral Activation of Macrophages in Human and Murine Sarcoma. Cancer Res..

[53] Kashfi K. (2020). Nitric oxide in cancer and beyond. Biochem. Pharmacol..

[54] Chan E.D., Riches D.W.H. (2001). IFN-γ + LPS induction of iNOS is modulated by ERK, JNK/SAPK, and p38 *^mapk^* in a mouse macrophage cell line. Am. J. Physiol. Cell. Physiol..

[55] Zhou P., Li Q., Su S., Dong W., Zong S., Ma Q., Yang X., Zuo D., Zheng S., Meng X. (2020). Interleukin 37 Suppresses M1 Macrophage Polarization Through Inhibition of the Notch1 and Nuclear Factor Kappa B Pathways. Front. Cell Dev. Biol..

[56] Wang X., Yuwen T.-J., Zhong Y., Li Z.-G., Wang X.-Y. (2023). A new method for predicting the prognosis of colorectal cancer patients through a combination of multiple tumor-associated macrophage markers at the invasive front. Heliyon.

[57] Peach R.J., Bajorath J., Naemura J., Leytze G., Greene J., Aruffo A., Linsley P.S. (1995). Both Extracellular Immunoglobin-like domains of CD80 contain residues critical for binding T cell surface receptors CTLA-4 and CD28. J. Biol. Chem..

[58] Yamaguchi T., Fushida S., Yamamoto Y., Tsukada T., Kinoshita J., Oyama K., Miyashita T., Tajima H., Ninomiya I., Munesue S. (2016). Tumor-associated macrophages of the M2 phenotype contribute to progression in gastric cancer with peritoneal dissemination. Gastric Cancer.

[59] You Y., Tian Z., Du Z., Wu K., Xu G., Dai M., Wang Y., Xiao M. (2022). M1-like tumor-associated macrophages cascade a mesenchymal/stem-like phenotype of oral squamous cell carcinoma via the IL6/Stat3/THBS1 feedback loop. J. Exp. Clin. Cancer Res..

[60] Zhang J., Li S., Liu F., Yang K. (2022). Role of CD68 in tumor immunity and prognosis prediction in pan-cancer. Sci. Rep..

[61] A Pulford K., Rigney E.M., Micklem K.J., Jones M., Stross W.P., Gatter K.C., Mason D.Y. (1989). KP1: A new monoclonal antibody that detects a monocyte/macrophage associated antigen in routinely processed tissue sections. J. Clin. Pathol..

[62] Chistiakov D.A., Killingsworth M.C., Myasoedova V.A., Orekhov A.N., Bobryshev Y.V. (2017). CD68/macrosialin: Not just a histochemical marker. Lab. Investig..

[63] Rashed R., Zaki M., Mohamed N., Mansou O., Refaey F. (2021). Prognostic Value of Tumor Associated Macrophage Markers CD163 and CD68 Immunohistochemistry in Classical Hodgkin Lymphoma. Clin. Lab..

[64] He Y., Dong Y., Zhang X., Ding Z., Song Y., Huang X., Chen S., Wang Z., Ni Y., Ding L. (2022). Lipid Droplet-Related PLIN2 in CD68+ Tumor-Associated Macrophage of Oral Squamous Cell Carcinoma: Implications for Cancer Prognosis and Immunotherapy. Front. Oncol..

[65] Kayal S., Mathur S., Karak A.K., Kumar L., Sharma A., Bakhshi S., Raina V. (2014). CD68 tumor-associated macrophage marker is not prognostic of clinical outcome in classical Hodgkin lymphoma. Leuk. Lymphoma.

[66] Buddingh E.P., Kuijjer M.L., Duim R.A., Bürger H., Agelopoulos K., Myklebost O., Serra M., Mertens F., Hogendoorn P.C., Lankester A.C. (2011). Tumor-infiltrating macrophages are associated with metastasis suppression in high-grade osteosarcoma: A rationale for treatment with macrophage activating agents. Clin. Cancer Res..

[67] Gomez-Brouchet A., Gilhodes J., Van Acker N., Brion R., Bouvier C., Assemat P., Gaspar N., Aubert S., Guinebretiere J.-M., Marie B. (2021). Characterization of Macrophages and Osteoclasts in the Osteosarcoma Tumor Microenvironment at Diagnosis: New Perspective for Osteosarcoma Treatment?. Cancers.

[68] Holness C.L., Simmons D.L. (1993). Molecular cloning of CD68, a human macrophage marker related to lysosomal glycoproteins. Blood.

[69] Medrek C., Pontén F., Jirström K., Leandersson K. (2012). The presence of tumor associated macrophages in tumor stroma as a prognostic marker for breast cancer patients. BMC Cancer.

[70] Kristiansen M., Graversen J.H., Jacobsen C., Sonne O., Hoffman H.-J., Law S.K.A., Moestrup S.K. (2001). Identification of the haemoglobin scavenger receptor. Nature.

[71] Komohara Y., Jinushi M., Takeya M. (2014). Clinical significance of macrophage heterogeneity in human malignant tumors. Cancer Sci..

[72] Wang J., Wu W., Yuan T., Wang L., Zang L., Liu Q., Wang L., Huo X., Huo B., Tang Y. (2024). Tumor-associated macrophages and PD-L1 in prostate cancer: A possible key to unlocking immunotherapy efficacy. Aging.

[73] Wei C., Yang C., Wang S., Shi D., Zhang C., Lin X., Liu Q., Dou R., Xiong B. (2019). Crosstalk between cancer cells and tumor associated macrophages is required for mesenchymal circulating tumor cell-mediated colorectal cancer metastasis. Mol. Cancer.

[74] Xie T., Fu D.-J., Li Z.-M., Lv D.-J., Song X.-L., Yu Y.-Z., Wang C., Li K.-J., Zhai B., Wu J. (2023). Correction: CircSMARCC1 facilitates tumor progression by disrupting the crosstalk between prostate cancer cells and tumor-associated macrophages via miR-1322/CCL20/CCR6 signaling. Mol. Cancer.

[75] Bao D., Zhao J., Zhou X., Yang Q., Chen Y., Zhu J., Yuan P., Yang J., Qin T., Wan S. (2019). Mitochondrial fission-induced mtDNA stress promotes tumor-associated macrophage infiltration and HCC progression. Oncogene.

[76] Yoshida C., Kadota K., Yamada K., Fujimoto S., Ibuki E., Ishikawa R., Haba R., Yokomise H. (2022). Tumor-associated CD163+ macrophage as a predictor of tumor spread through air spaces and with CD25+ lymphocyte as a prognostic factor in resected stage I lung adenocarcinoma. Lung Cancer.

[77] Guo F., Feng Y.-C., Zhao G., Zhang R., Cheng Z.-Z., Kong W.-N., Wu H.-L., Xu B., Lv X., Ma X.-M. (2020). Tumor-Associated CD163+ M2 Macrophage Infiltration is Highly Associated with PD-L1 Expression in Cervical Cancer. Cancer Manag. Res..

[78] Ramos R.N., Rodriguez C., Hubert M., Ardin M., Treilleux I., Ries C.H., Lavergne E., Chabaud S., Colombe A., Trédan O. (2020). CD163^+^ tumor-associated macrophage accumulation in breast cancer patients reflects both local differentiation signals and systemic skewing of monocytes. Clin. Transl. Immunol..

[79] Liang Y., Lei Y., Liang M., Du M., Liu Z., Li X., Meng X., Zhou B., Gao Y. (2021). GBE1 Is an Independent Prognostic Marker and Associated With CD163+ Tumor-Associated Macrophage Infiltration in Lung Adenocarcinoma. Front. Oncol..

[80] Zhu D., Shi X., Tian Y., Li H., Tang B., Zhang Z., Zhang Z., Zuo L. (2023). Combining expression of RNF43 and infiltration level of CD163^+^ tumor associated macrophage predicts prognosis of clear cell renal cell carcinoma. Cancer Med..

[81] Edin S., Wikberg M.L., Dahlin A.M., Rutegård J., Öberg Å., Oldenborg P.-A., Palmqvist R. (2012). The distribution of macrophages with a M1 or M2 phenotype in relation to prognosis and the molecular characteristics of colorectal cancer. PLoS ONE.

[82] Nyström H., Jönsson M., Nilbert M., Carneiro A. (2023). Immune-cell infiltration in high-grade soft tissue sarcomas; prognostic implications of tumor-associated macrophages and B-cells. Acta Oncol..

[83] Sun Y., Xu S. (2018). Tumor-Associated CD204-Positive Macrophage Is a Prognostic Marker in Clinical Stage I Lung Adenocarcinoma. BioMed Res. Int..

[84] He Y., Zhou S., Deng F., Zhao S., Chen W., Wang D., Chen X., Hou J., Zhang J., Zhang W. (2019). Clinical and transcriptional signatures of human CD204 reveal an applicable marker for the protumor phenotype of tumor-associated macrophages in breast cancer. Aging.

[85] Miyasato Y., Shiota T., Ohnishi K., Pan C., Yano H., Horlad H., Yamamoto Y., Yamamoto-Ibusuki M., Iwase H., Takeya M. (2017). High density of CD204-positive macrophages predicts worse clinical prognosis in patients with breast cancer. Cancer Sci..

[86] Xie Y., Tang G., Xie P., Zhao X., Chen C., Li X., Zhang Y., Wang B., Luo Y. (2024). High CD204^+^ tumor-associated macrophage density predicts a poor prognosis in patients with clear cell renal cell carcinoma. J. Cancer.

[87] Li Z., Maeda D., Yoshida M., Umakoshi M., Nanjo H., Shiraishi K., Saito M., Kohno T., Konno H., Saito H. (2018). The intratumoral distribution influences the prognostic impact of CD68- and CD204-positive macrophages in non-small cell lung cancer. Lung Cancer.

[88] Ikarashi D., Kitano S., Tsuyukubo T., Takenouchi K., Nakayama T., Onagi H., Sakaguchi A., Yamashita M., Mizugaki H., Maekawa S. (2022). Pretreatment tumour immune microenvironment predicts clinical response and prognosis of muscle-invasive bladder cancer in the neoadjuvant chemotherapy setting. Br. J. Cancer.

[89] Ichimura T., Morikawa T., Kawai T., Nakagawa T., Matsushita H., Kakimi K., Kume H., Ishikawa S., Homma Y., Fukayama M. (2014). Prognostic significance of CD204-positive macrophages in upper urinary tract cancer. Ann. Surg. Oncol..

[90] Qi Y.-Q., Xiong F., Chen Y.-J. (2023). The Correlation between tumor-associated macrophages and the prognosis of east asian hepatocellular carcinoma patients: A systematic review and meta-analysis. Pathol. Res. Pr..

[91] Geijtenbeek T.B., Kwon D.S., Torensma R., van Vliet S.J., van Duijnhoven G.C., Middel J., Cornelissen I.L., Nottet H.S., KewalRamani V.N., Littman D.R. (2000). DC-SIGN, a dendritic cell–specific HIV-1-binding protein that enhances trans-infection of T cells. Cell.

[92] Hu B., Wang Z., Zeng H., Qi Y., Chen Y., Wang T., Wang J., Chang Y., Bai Q., Xia Y. (2020). Blockade of DC-SIGN+ Tumor-Associated Macrophages Reactivates Antitumor Immunity and Improves Immunotherapy in Muscle-Invasive Bladder Cancer. Cancer Res..

[93] Yamada R., Ohnishi K., Pan C., Yano H., Fujiwara Y., Shiota T., Mikami Y., Komohara Y. (2023). Expression of macrophage/dendritic cell–related molecules in lymph node sinus macrophages. Microbiol. Immunol..

[94] He M., Jiang X., Miao J., Feng W., Xie T., Liao S., Qin Z., Tang H., Lin C., Li B. (2023). A new insight of immunosuppressive microenvironment in osteosarcoma lung metastasis. Exp. Biol. Med..

[95] Graziano F., Vicenzi E., Poli G. (2016). Plastic restriction of HIV-1 replication in human macrophages derived from M1/M2 polarized monocytes. J. Leukoc. Biol..

[96] Ugel S., De Sanctis F., Mandruzzato S., Bronte V. (2015). Tumor-induced myeloid deviation: When myeloid-derived suppressor cells meet tumor-associated macrophages. J. Clin. Investig..

[97] Zlotnik A., Yoshie O. (2012). The Chemokine Superfamily Revisited. Immunity.

[98] Deshmane S.L., Kremlev S., Amini S., Sawaya B.E. (2009). Monocyte Chemoattractant Protein-1 (MCP-1): An overview. J. Interf. Cytokine Res..

[99] Qian B.-Z., Li J., Zhang H., Kitamura T., Zhang J., Campion L.R., Kaiser E.A., Snyder L.A., Pollard J.W. (2011). CCL2 recruits inflammatory monocytes to facilitate breast-tumour metastasis. Nature.

[100] Cho H.R., Kumari N., Vu H.T., Kim H., Park C.-K., Choi S.H. (2019). Increased Antiangiogenic Effect by Blocking CCL2-dependent Macrophages in a Rodent Glioblastoma Model: Correlation Study with Dynamic Susceptibility Contrast Perfusion MRI. Sci. Rep..

[101] Zheng Y., Wang Z., Wei S., Liu Z., Chen G. (2021). Epigenetic silencing of chemokine CCL2 represses macrophage infiltration to potentiate tumor development in small cell lung cancer. Cancer Lett..

[102] Cranford T.L., Velázquez K.T., Enos R.T., Bader J.E., Carson M.S., Chatzistamou I., Nagarkatti M., Murphy E.A. (2017). Loss of monocyte chemoattractant protein-1 expression delays mammary tumorigenesis and reduces localized inflammation in the C3(1)/SV40Tag triple negative breast cancer model. Cancer Biol. Ther..

[103] Rogic A., Pant I., Grumolato L., Fernandez-Rodriguez R., Edwards A., Das S., Sun A., Yao S., Qiao R., Jaffer S. (2021). High endogenous CCL2 expression promotes the aggressive phenotype of human inflammatory breast cancer. Nat. Commun..

[104] Moisan F., Francisco E.B., Brozovic A., Duran G.E., Wang Y.C., Chaturvedi S., Seetharam S., Snyder L.A., Doshi P., Sikic B.I. (2014). Enhancement of paclitaxel and carboplatin therapies by CCL2 blockade in ovarian cancers. Mol. Oncol..

[105] Liu J.-F., Chen P.-C., Chang T.-M., Hou C.-H. (2020). Monocyte Chemoattractant Protein-1 promotes cancer cell migration via c-Raf/MAPK/AP-1 pathway and MMP-9 production in osteosarcoma. J. Exp. Clin. Cancer Res..

[106] Ohba T., A Cole H., Cates J.M., A Slosky D., Haro H., Ando T., Schwartz H.S., Schoenecker J.G. (2014). Bisphosphonates inhibit osteosarcoma-mediated osteolysis via attenuation of tumor expression of MCP-1 and RANKL. J. Bone Miner. Res..

[107] Chen P., Zhou J., Li J., Zhang Q., Zuo Q. (2019). TIPE1 suppresses osteosarcoma tumor growth by regulating macrophage infiltration. Clin. Transl. Oncol..

[108] Zeng Z., Lan T., Wei Y., Wei X. (2022). CCL5/CCR5 axis in human diseases and related treatments. Genes Dis..

[109] Huang R., Wang S., Wang N., Zheng Y., Zhou J., Yang B., Wang X., Zhang J., Guo L., Wang S. (2020). CCL5 derived from tumor-associated macrophages promotes prostate cancer stem cells and metastasis via activating β-catenin/STAT3 signaling. Cell Death Dis..

[110] Yonemitsu K., Miyasato Y., Shiota T., Shinchi Y., Fujiwara Y., Hosaka S., Yamamoto Y., Komohara Y. (2021). Soluble Factors Involved in Cancer Cell–Macrophage Interaction Promote Breast Cancer Growth. Anticancer. Res..

[111] Nie Y., Huang H., Guo M., Chen J., Wu W., Li W., Xu X., Lin X., Fu W., Yao Y.-D. (2019). Breast Phyllodes Tumors Recruit and Repolarize Tumor-Associated Macrophages via Secreting CCL5 to Promote Malignant Progression, Which Can Be Inhibited by CCR5 Inhibition Therapy. Clin. Cancer Res..

[112] Terashima Y., Toda E., Itakura M., Otsuji M., Yoshinaga S., Okumura K., Shand F.H.W., Komohara Y., Takeda M., Kokubo K. (2020). Targeting FROUNT with disulfiram suppresses macrophage accumulation and its tumor-promoting properties. Nat. Commun..

[113] Ma J., Shayiti F., Ma J., Wei M., Hua T., Zhang R., Su J., Chen P. (2021). Tumor-associated macrophage-derived CCL5 promotes chemotherapy resistance and metastasis in prostatic cancer. Cell Biol. Int..

[114] Wang S.-W., Wu H.-H., Liu S.-C., Wang P.-C., Ou W.-C., Chou W.-Y., Shen Y.-S., Tang C.-H. (2012). CCL5 and CCR5 Interaction Promotes Cell Motility in Human Osteosarcoma. PLoS ONE.

[115] Wang S.-W., Liu S.-C., Sun H.-L., Huang T.-Y., Chan C.-H., Yang C.-Y., Yeh H.-I., Huang Y.-L., Chou W.-Y., Lin Y.-M. (2015). CCL5/CCR5 axis induces vascular endothelial growth factor-mediated tumor angiogenesis in human osteosarcoma microenvironment. Carcinogenesis.

[116] Islam S.A., Ling M.F., Leung J., Shreffler W.G., Luster A.D. (2013). Identification of human CCR8 as a CCL18 receptor. J. Exp. Med..

[117] Sui X., Chen C., Zhou X., Wen X., Shi C., Chen G., Liu J., He Z., Yao Y., Li Y. (2023). Integrative analysis of bulk and single-cell gene expression profiles to identify tumor-associated macrophage-derived CCL18 as a therapeutic target of esophageal squamous cell carcinoma. J. Exp. Clin. Cancer Res..

[118] Lin L., Chen Y.-S., Yao Y.-D., Chen J.-Q., Chen J.-N., Huang S.-Y., Zeng Y.-J., Yao H.-R., Zeng S.-H., Fu Y.-S. (2015). CCL18 from tumor-associated macrophages promotes angiogenesis in breast cancer. Oncotarget.

[119] Chen J., Yao Y., Gong C., Yu F., Su S., Chen J., Liu B., Deng H., Wang F., Lin L. (2011). CCL18 from Tumor-Associated Macrophages Promotes Breast Cancer Metastasis via PITPNM3. Cancer Cell.

[120] Su Y., Zhou Y., Sun Y.-J., Wang Y.-L., Yin J.-Y., Huang Y.-J., Zhang J.-J., He A.-N., Han K., Zhang H.-Z. (2019). Macrophage-derived CCL18 promotes osteosarcoma proliferation and migration by upregulating the expression of UCA1. J. Mol. Med..

[121] Li C., Xiang F., Gong Y., Fu Y., Chen G., Wang Z., Li Z., Wei D. (2024). Tumor-derived microparticles promoted M2-like macrophages polarization to stimulate osteosarcoma progression. Int. J. Biochem. Cell Biol..

[122] Chen J., Zhao D., Zhang L., Zhang J., Xiao Y., Wu Q., Wang Y., Zhan Q. (2022). Tumor-associated macrophage (TAM)-derived CCL22 induces FAK addiction in esophageal squamous cell carcinoma (ESCC). Cell. Mol. Immunol..

[123] Tsujikawa T., Yaguchi T., Ohmura G., Ohta S., Kobayashi A., Kawamura N., Fujita T., Nakano H., Shimada T., Takahashi T. (2013). Autocrine and paracrine loops between cancer cells and macrophages promote lymph node metastasis via CCR4/CCL22 in head and neck squamous cell carcinoma. Int. J. Cancer.

[124] Wolf-Dennen K., Gordon N., Kleinerman E.S. (2020). Exosomal communication by metastatic osteosarcoma cells modulates alveolar macrophages to an M2 tumor-promoting phenotype and inhibits tumoricidal functions. OncoImmunology.

[125] Zhang Y., Chen Y., Chen C., Guo H., Zhou C., Wang H., Liu Z. (2023). PITX1 suppresses osteosarcoma metastasis through exosomal LINC00662-mediated M2 macrophage polarization. Clin. Exp. Metastasis.

[126] Korbecki J., Bosiacki M., Szatkowska I., Kupnicka P., Chlubek D., Baranowska-Bosiacka I. (2024). The Clinical Significance and Involvement in Molecular Cancer Processes of Chemokine CXCL1 in Selected Tumors. Int. J. Mol. Sci..

[127] Miyake M., Hori S., Morizawa Y., Tatsumi Y., Nakai Y., Anai S., Torimoto K., Aoki K., Tanaka N., Shimada K. (2016). Mediated Interaction of Cancer Cells with Tumor-Associated Macrophages and Cancer-Associated Fibroblasts Promotes Tumor Progression in Human Bladder Cancer. Neoplasia.

[128] Zhang L., Gu S., Wang L., Zhao L., Li T., Zhao X., Zhang L. (2024). M2 macrophages promote PD-L1 expression in triple-negative breast cancer via secreting CXCL1. Pathol. Res. Pr..

[129] Lee C.-W., Chiang Y.-C., Yu P.-A., Peng K.-T., Chi M.-C., Lee M.-H., Fang M.-L., Lee K.-H., Hsu L.-F., Liu J.-F. (2021). A Role of CXCL1 Drives Osteosarcoma Lung Metastasis via VCAM-1 Production. Front. Oncol..

[130] Chao C.-C., Lee C.-W., Chang T.-M., Chen P.-C., Liu J.-F. (2020). CXCL1/CXCR2 Paracrine Axis Contributes to Lung Metastasis in Osteosarcoma. Cancers.

[131] Chang M.S., McNinch J., Basu R., Simonet S. (1994). Cloning and characterization of the human neutrophil-activating peptide (ENA-78) gene. J. Biol. Chem..

[132] Su P., Jiang L., Zhang Y., Yu T., Kang W., Liu Y., Yu J. (2022). Crosstalk between tumor-associated macrophages and tumor cells promotes chemoresistance via CXCL5/PI3K/AKT/mTOR pathway in gastric cancer. Cancer Cell Int..

[133] Dang H., Wu W., Wang B., Cui C., Niu J., Chen J., Chen Z., Liu Y. (2017). CXCL5 Plays a Promoting Role in Osteosarcoma Cell Migration and Invasion in Autocrine- and Paracrine-Dependent Manners. Oncol. Res..

[134] Proost P., Wuyts A., Conings R., Lenaerts J.P., Billiau A., Opdenakker G., Van Damme J. (1993). Human and bovine granulocyte chemotactic protein-2: Complete amino acid sequence and functional characterization as chemokines. Biochemistry.

[135] Lee H.-L., Tsai Y.-C., Pikatan N.W., Yeh C.-T., Yadav V.K., Chen M.-Y., Tsai J.-T. (2023). Tumor-Associated Macrophages Affect the Tumor Microenvironment and Radioresistance via the Upregulation of CXCL6/CXCR2 in Hepatocellular Carcinoma. Biomedicines.

[136] Liu G., An L., Zhang H., Du P., Sheng Y. (2019). Activation of CXCL6/CXCR1/2 Axis Promotes the Growth and Metastasis of Osteosarcoma Cells in vitro and in vivo. Front. Pharmacol..

[137] Knall C., Worthen G.S., Johnson G.L. (1997). Interleukin 8-stimulated phosphatidylinositol-3-kinase activity regulates the migration of human neutrophils independent of extracellular signal-regulated kinase and p38 mitogen-activated protein kinases. Proc. Natl. Acad. Sci. USA.

[138] Brat D.J., Bellail A.C., Van Meir E.G. (2005). The role of interleukin-8 and its receptors in gliomagenesis and tumoral angiogenesis. Neuro-Oncol..

[139] Wu H., Zhang X., Han D., Cao J., Tian J. (2020). Tumour-associated macrophages mediate the invasion and metastasis of bladder cancer cells through CXCL8. PeerJ.

[140] Fang W., Ye L., Shen L., Cai J., Huang F., Wei Q., Fei X., Chen X., Guan H., Wang W. (2014). Tumor-associated macrophages promote the metastatic potential of thyroid papillary cancer by releasing CXCL8. Carcinogenesis.

[141] Piao H., Fu L., Wang Y., Liu Y., Wang Y., Meng X., Yang D., Xiao X., Zhang J. (2022). A positive feedback loop between gastric cancer cells and tumor-associated macrophage induces malignancy progression. J. Exp. Clin. Cancer Res..

[142] Hosono M., Koma Y.-I., Takase N., Urakawa N., Higashino N., Suemune K., Kodaira H., Nishio M., Shigeoka M., Kakeji Y. (2017). CXCL8 derived from tumor-associated macrophages and esophageal squamous cell carcinomas contributes to tumor progression by promoting migration and invasion of cancer cells. Oncotarget.

[143] Nie G., Cao X., Mao Y., Lv Z., Lv M., Wang Y., Wang H., Liu C. (2021). Tumor-associated macrophages-mediated CXCL8 infiltration enhances breast cancer metastasis: Suppression by Danirixin. Int. Immunopharmacol..

[144] Jiang H., Wang X., Miao W., Wang B., Qiu Y. (2017). CXCL8 promotes the invasion of human osteosarcoma cells by regulation of PI3K/Akt signaling pathway. APMIS.

[145] Hirano T., Yasukawa K., Harada H., Taga T., Watanabe Y., Matsuda T., Kashiwamura S.-I., Nakajima K., Koyama K., Iwamatsu A. (1986). Complementary DNA for a novel human interleukin (BSF-2) that induces B lymphocytes to produce immunoglobulin. Nature.

[146] Zhang L., Xu Y., Sun J., Chen W., Zhao L., Ma C., Wang Q., Sun J., Huang B., Zhang Y. (2017). M2-like tumor-associated macrophages drive vasculogenic mimicry through amplification of IL-6 expression in glioma cells. Oncotarget.

[147] Lin Y.-M., Chang Z.-L., Liao Y.-Y., Chou M.-C., Tang C.-H. (2013). IL-6 promotes ICAM-1 expression and cell motility in human osteosarcoma. Cancer Lett..

[148] Tu B., Du L., Fan Q.-M., Tang Z., Tang T.-T. (2012). STAT3 activation by IL-6 from mesenchymal stem cells promotes the proliferation and metastasis of osteosarcoma. Cancer Lett..

[149] Vane J.R., Bakhle Y.S., Botting R.M. (1998). Cyclooxygenases 1 and 2. Annu. Rev. Pharmacol. Toxicol..

[150] Qin Q., Ji H., Li D., Zhang H., Zhang Z., Zhang Q. (2021). Tumor-associated macrophages increase COX-2 expression promoting endocrine resistance in breast cancer via the PI3K/Akt/mTOR pathway. Neoplasma.

[151] Tsai C.-S., Chen F.-H., Wang C.-C., Huang H.-L., Jung S.-M., Wu C.-J., Lee C.-C., McBride W.H., Chiang C.-S., Hong J.-H. (2007). Macrophages From Irradiated Tumors Express Higher Levels of iNOS, Arginase-I and COX-2, and Promote Tumor Growth. Int. J. Radiat. Oncol..

[152] Han Y., Guo W., Ren T., Huang Y., Wang S., Liu K., Zheng B., Yang K., Zhang H., Liang X. (2019). Tumor-associated macrophages promote lung metastasis and induce epithelial-mesenchymal transition in osteosarcoma by activating the COX-2/STAT3 axis. Cancer Lett..

[153] Bassiouni W., Ali M.A.M., Schulz R. (2021). Multifunctional intracellular matrix metalloproteinases: Implications in disease. FEBS J..

[154] Wang X., Liang J., Koike T., Sun H., Ichikawa T., Kitajima S., Morimoto M., Shikama H., Watanabe T., Sasaguri Y. (2004). Overexpression of human matrix metalloproteinase-12 enhances the development of inflammatory arthritis in transgenic rabbits. Am. J. Pathol..

[155] Kerzeli I.K., Kostakis A., Türker P., Malmström P.-U., Hemdan T., Mezheyeuski A., Ward D.G., Bryan R.T., Segersten U., Lord M. (2023). Elevated levels of MMP12 sourced from macrophages are associated with poor prognosis in urothelial bladder cancer. BMC Cancer.

[156] Zhou Q., Xian M., Xiang S., Xiang D., Shao X., Wang J., Cao J., Yang X., Yang B., Ying M. (2017). All-Trans Retinoic Acid Prevents Osteosarcoma Metastasis by Inhibiting M2 Polarization of Tumor-Associated Macrophages. Cancer Immunol. Res..

[157] Fujikawa M., Koma Y.-I., Hosono M., Urakawa N., Tanigawa K., Shimizu M., Kodama T., Sakamoto H., Nishio M., Shigeoka M. (2021). Chemokine (C-C Motif) Ligand 1 Derived from Tumor-Associated Macrophages Contributes to Esophageal Squamous Cell Carcinoma Progression via CCR8-Mediated Akt/Proline-Rich Akt Substrate of 40 kDa/Mammalian Target of Rapamycin Pathway. Am. J. Pathol..

[158] Liu W., Wang W., Wang X., Xu C., Zhang N., Di W. (2020). Cisplatin-stimulated macrophages promote ovarian cancer migration via the CCL20-CCR6 axis. Cancer Lett..

[159] Kadomoto S., Izumi K., Hiratsuka K., Nakano T., Naito R., Makino T., Iwamoto H., Yaegashi H., Shigehara K., Kadono Y. (2019). Tumor-Associated Macrophages Induce Migration of Renal Cell Carcinoma Cells via Activation of the CCL20-CCR6 Axis. Cancers.

[160] Zhou K., Cheng T., Zhan J., Peng X., Zhang Y., Wen J., Chen X., Ying M. (2020). Targeting tumor-associated macrophages in the tumor microenvironment. Oncol. Lett..

[161] Zhang D., Shi R., Xiang W., Kang X., Tang B., Li C., Gao L., Zhang X., Zhang L., Dai R. (2020). The Agpat4/LPA axis in colorectal cancer cells regulates antitumor responses via p38/p65 signaling in macrophages. Signal Transduct. Target. Ther..

[162] Anand N., Peh K.H., Kolesar J.M. (2023). Macrophage Repolarization as a Therapeutic Strategy for Osteosarcoma. Int. J. Mol. Sci..

[163] Guan W., Li F., Zhao Z., Zhang Z., Hu J., Zhang Y. (2021). Tumor-Associated Macrophage Promotes the Survival of Cancer Cells upon Docetaxel Chemotherapy via the CSF1/CSF1R–CXCL12/CXCR4 Axis in Castration-Resistant Prostate Cancer. Genes.

[164] Liang X., Guo W., Ren T., Huang Y., Sun K., Zhang H., Yu Y., Wang W., Niu J. (2020). Macrophages reduce the sensitivity of osteosarcoma to neoadjuvant chemotherapy drugs by secreting Interleukin-1 beta. Cancer Lett..

[165] Kimura Y., Sumiyoshi M. (2015). Antitumor and antimetastatic actions of dihydroxycoumarins (esculetin or fraxetin) through the inhibition of M2 macrophage differentiation in tumor-associated macrophages and/or G1 arrest in tumor cells. Eur. J. Pharmacol..

