# Supplementary material for: Surface Markers and Chemokines/Cytokines of Tumor-Associated Macrophages in Osteosarcoma and Other Carcinoma Microenviornments—Contradictions and Comparisons
Source: Cancers (Basel). 2024 Aug 8;16(16):2801. doi: 10.3390/cancers16162801 (PMC11353089; doi:10.3390/cancers16162801)
Supplement: Supplementary file 1 [file cancers-16-02801-s001.zip › cancers-3109443-supplementary.pdf]

Supplementary Materials

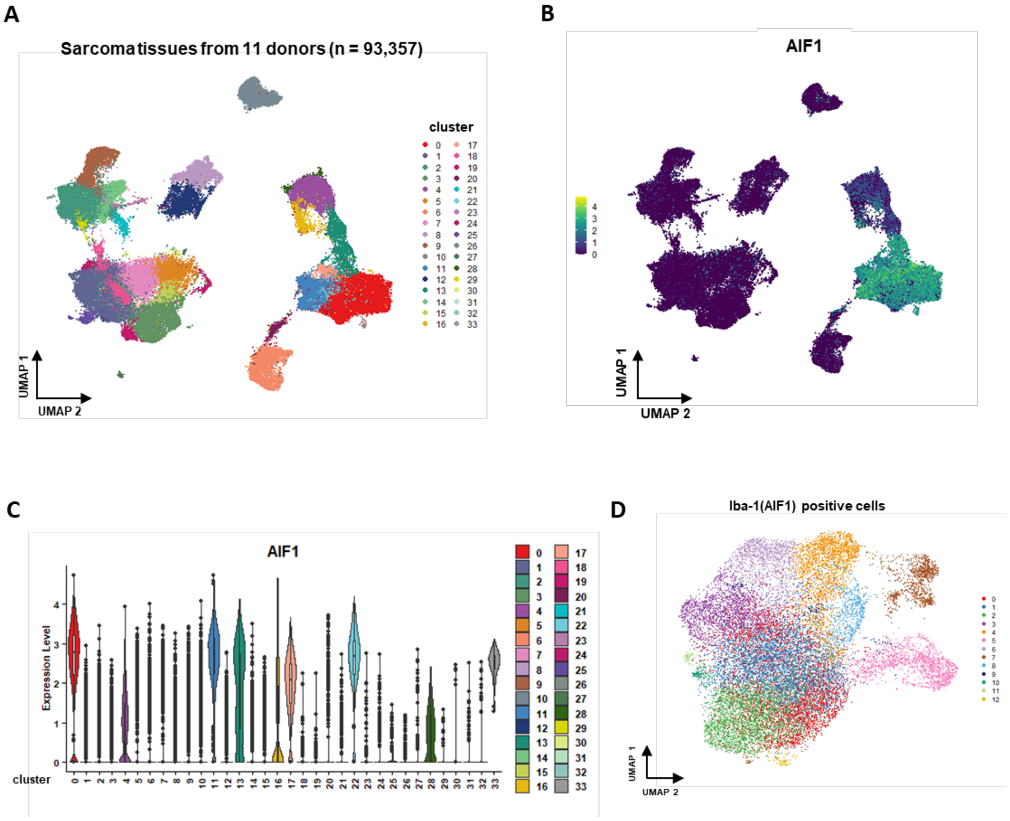

Figure S1. (**a**) UMAP plot of the cells (n = 93,357) from 11 osteosarcoma tissues (GSE152048, primary, n = 7, recurrent, n = 2, and lung metastatic, n = 2) clustered into 34 clusters. (**b**) UMAP plot of the expression of gene AIF1 (Iba-1) across all the cells. (**c**) Violin plot of the expression of gene AIF1 (Iba-1) across all 34 clusters. (**d**) UMAP plot of the selected AIF1 (Iba-1) positive cells (included cluster 0, 11, 17, 22, 33, and part of 13 which is positive for AIF1). The AIF1(Iba-1) positive cells serve as myeloid lineage cells and re-clustered into 13 clusters.

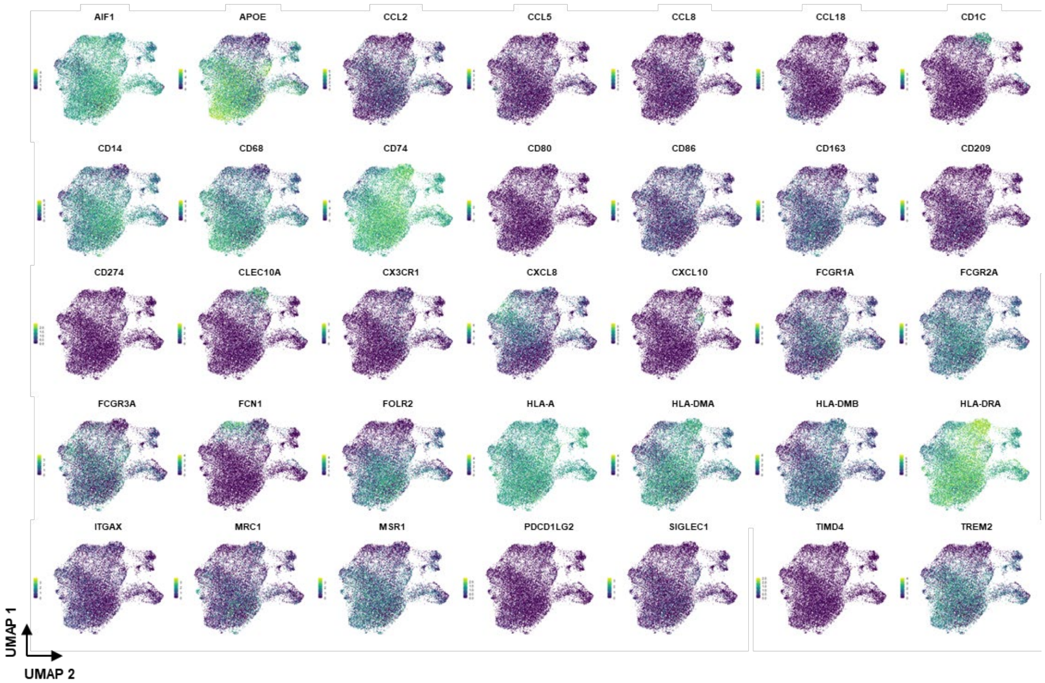

Figure S2. UMAP plots of the expression of the indicated genes in the AIF1(Iba-1) positive cells of all 11 osteosarcoma tissues.

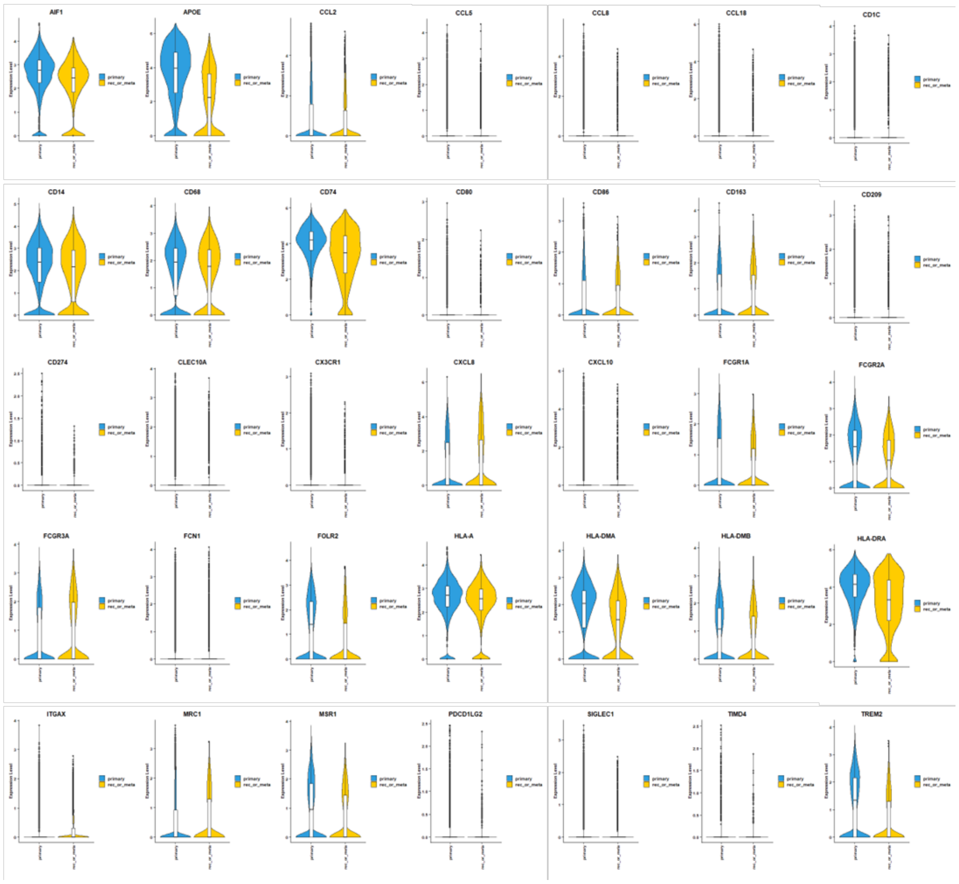

Figure S3. Violin plots of the expression of the indicated genes in the AIF1(Iba-1) positive cells across primary and metastatic/recurrent tissues.
